# Neisseria meningitidis serogroup W(P1.5-2) sepsis presenting with myopericarditis in an elderly previously healthy male

**DOI:** 10.1016/j.idcr.2021.e01238

**Published:** 2021-07-24

**Authors:** Michael Platten, Layth Aladellie

**Affiliations:** a Karolinska University Hospital, Stockholm, Sweden; b Karolinska Institutet, Stockholm, Sweden

**Keywords:** Neisseria meningitidis, Myopericarditis, Prophylaxis, Duodenal perforation

## Abstract

A rare complication of Neisseria meningitidis is pericarditis. Here a 74-year-old male with Neisseria meningitidis serogroup W(P1.5−2) presented with myopericarditis. The patient developed cardiac tamponade and a pericardiocentesis was subsequently performed. The patient also developed a duodenal perforation, possibly secondary to the stress from being critically ill. The patient fully recovered.

## Introduction

Neisseria meningitidis, a gram-negative diplococcus, most often affects young adults living in close quarters and commonly presents with meningitis or septicemia. The bacteria colonize the nasopharyngeal mucosa and it is estimated that 10 % of the population are asymptomatic carriers [1]. There are 5 serogroups of Neisseria meningitidis that account for the vast majority of cases: serogroup A, B, C, W, and Y [2]. There has been a steady increase in Neisseria meningitidis serogroup W (MenW) in Europe between 2013 and 2017, especially in the patient population aged 45 years or older [3]. Sub-Saharan Africa which has had high endemic Neisseria meningitidis rates over the past century, often referred to as the “meningitis belt of sub-Saharan Africa”, has seen a dramatic reduction in MenA cases after introduction of the conjugate MenA vaccine in 2010 [4]. Currently the dominating strains in the region include MenC (53 %), MenW (30 %), and MenX (13 %) [5]. A meta-analysis revealed the highest case fatality rate among serogroups W and C and that the fatality rate rose dramatically with age, reaching 32.2 % in 80-year-old patients [6]. A classification of pericarditis associated with Neisseria meningitidis was presented in 1997, where three distinct pathophysiological mechanisms were elucidated: Disseminated meningococcal disease with pericarditis, isolated meningococcal pericarditis, and reactive meningococcal pericarditis [7]. Previous cases are often young individuals that may develop pericarditis secondary to disseminated disease [8]. In this patient case, a 74-year-old male presented with myopericarditis and was subsequently found to have disseminated meningococcal disease.

## Case

A 74-year-old retired man born in South Asia who moved to Sweden in his early twenties, presented to the ER with increasing dyspnea and retrosternal pain. Two days prior to admission the patient began experiencing generalized muscle pain, a headache, and mild retrosternal pain. The day before admission he developed a slight increase in his retrosternal chest pain and experienced dyspnea while walking. On the day of admission, the patient had dyspnea at rest and a retrosternal pain that led him to seek emergency care. The patient denied any fever, vomiting, coughing, diarrhea, or changes to micturition and no one in his environment was ill at the time.

The patient lived a healthy life with daily exercise, a healthy diet, and no current diagnosis or treatment. The patient had been treated and cured of tuberculosis at the age of 23 in his home country. He was a non-smoker and besides consuming 4 centiliters of whiskey to dinner every evening, he consumed a small amount of the Swedish tobacco of the mouth called “snus”. He had no family history of cardiovascular events and his vaccination status was unknown.

**Fig. 1:**
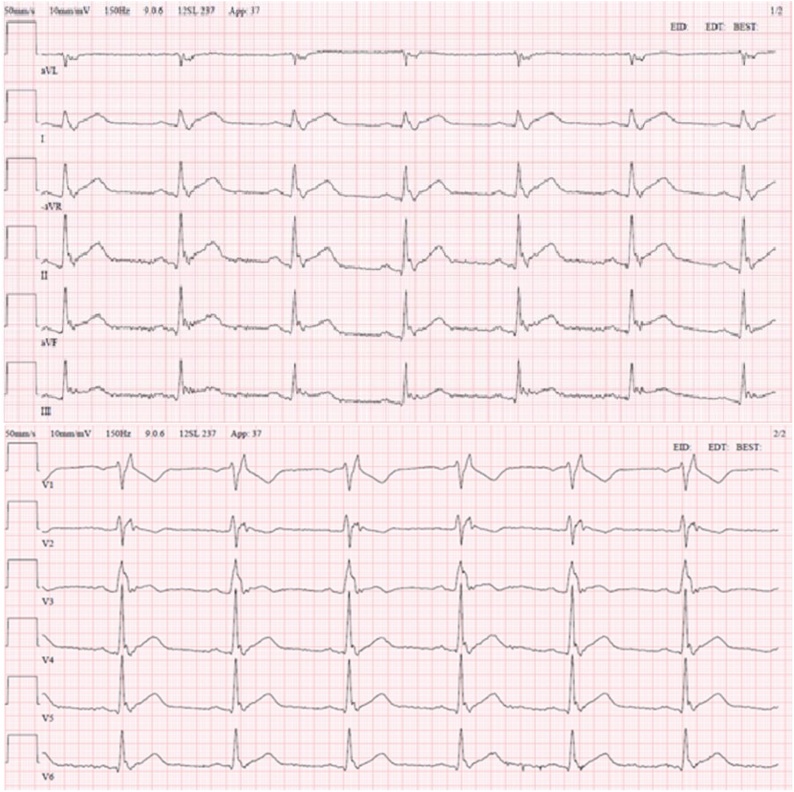
EKG at Admission. Initially in the ER, the EKG showed generalized ST-elevations as well as a right bundle branch block. There were no earlier EKG registrations available for comparison.

**Fig. 2:**
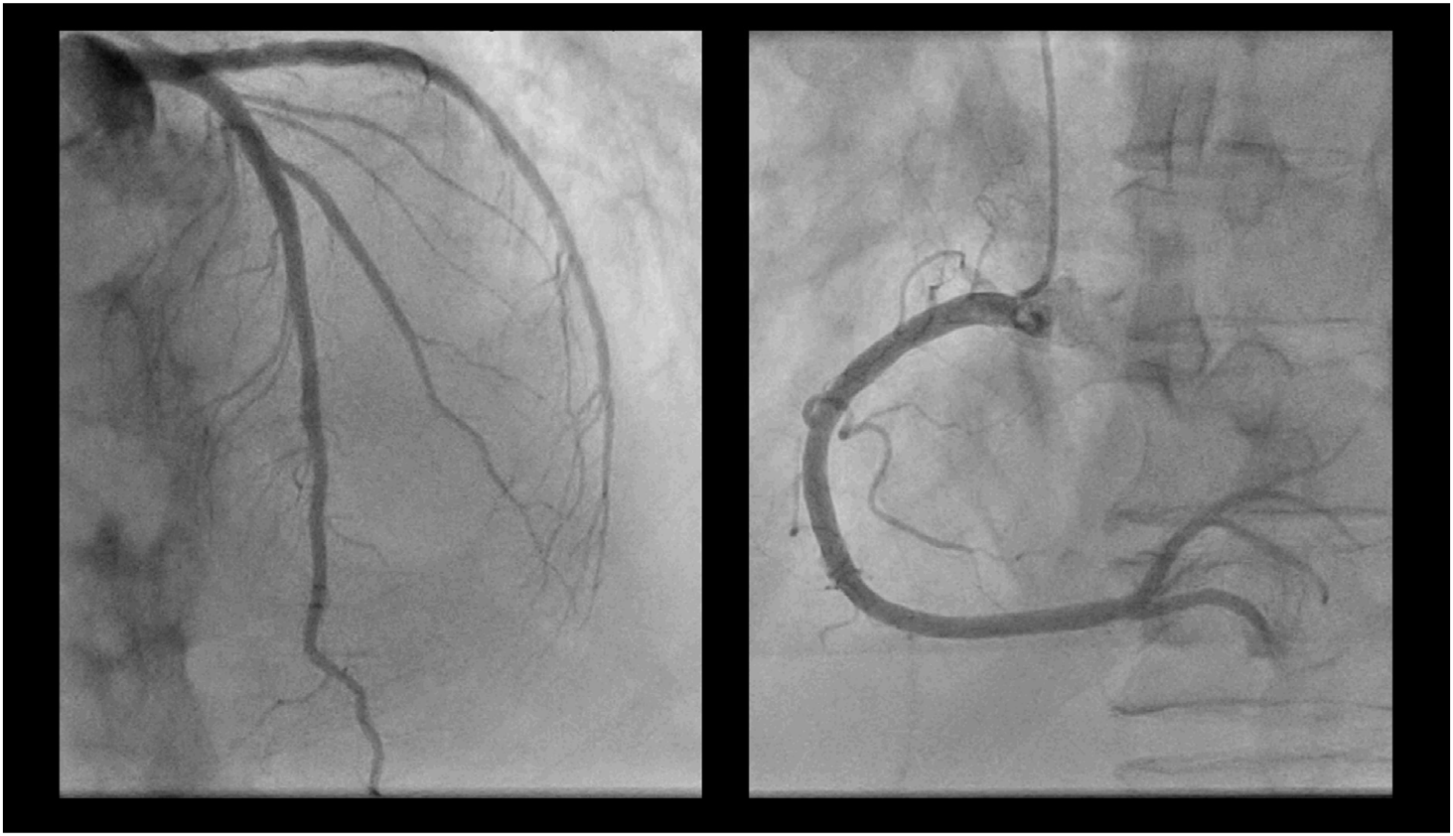
Coronary Angiography at Admission. The results of the coronary angiography showed no coronary artery obstruction.

The patient arrived at the ER and was evaluated according to *ABCDE* (Table 1). The patient’s breathing rate was slightly increased and his blood pressure was low. The blood tests at admission were strongly suggestive of an infection and incipient sepsis: Creatinine: 220 μmol/L (ref<100), CRP: 435 mg/L (ref<5), leukocytes: 14.5 10^9^/L (ref:3.5–5.5), troponin T: 172 ng/L (ref<5), lactate: 5 mmol/L (ref<2.3*).* This prompted bacterial cultures which later grew Neisseria meningitidis with a time to detection of 11.2 h (Table 2). Due to the retrosternal chest pain, an EKG was performed in the ER which showed generalized ST-elevations as well as a right bundle branch block (Fig. 1). The patient was rushed for a coronary angiography in order to rule out myocardial infarction (Fig. 2). A computed tomography scan was performed and showed no signs of lung embolus, pneumonia or damage indicative of his history of tuberculosis. There was however a small level of bilateral pleural effusion measuring 1.5 cm.

**Table 1:** *ABCDE* at Admission.

A	No obstruction.
B	Bilateral vesicular lung sounds. Saturation: 97 %. Breaths per minute: 20.
C	Pulse: 80 and regular. Blood Pressure: 93/54. Peripherally warm. Non-tender, soft abdomen
D	Glasgow coma scale: 15 points.
E	Temperature: 35.6 °C.

A: Airway, B: Breathing, C: Circulation, D: Disability, E: Exposure.

**Table 2:** Blood Culture and Resistance Pattern.

Blood Culture	
1	Positive for N. meningitidis^a^
2	Positive for N. meningitidis^a^
3	Positive for N. meningitidis^a^
4	Positive for N. meningitidis^a^

Nasopharynx	
1	Positive for N. meningitidis

Resistance Pattern for N. meningococcus	
S	Benzyl-penicillin
S	Meropenem
S	Chloramphenicol
S	Rifampicin
S	Cefotaxime
S	Ciprofloxacin

The patient was also negative for HIV 1 and 2.

S: Sensitive.

a Time to detection was 11.2 h and later verified to be N. meningitidis serogroup W, genotype P.1.5–2.

After the initial coronary angiography showed free flow and the laboratory values indicated an infection, the patient was placed on broad-spectrum antibiotics: piperacillin and tazobactam. When the blood cultures showed gram-negative cocci, the antibiotics were switched to cefotaxime for better coverage. When the resistance pattern arrived, the patient was switched to benzyl-penicillin.

**Fig. 3:**
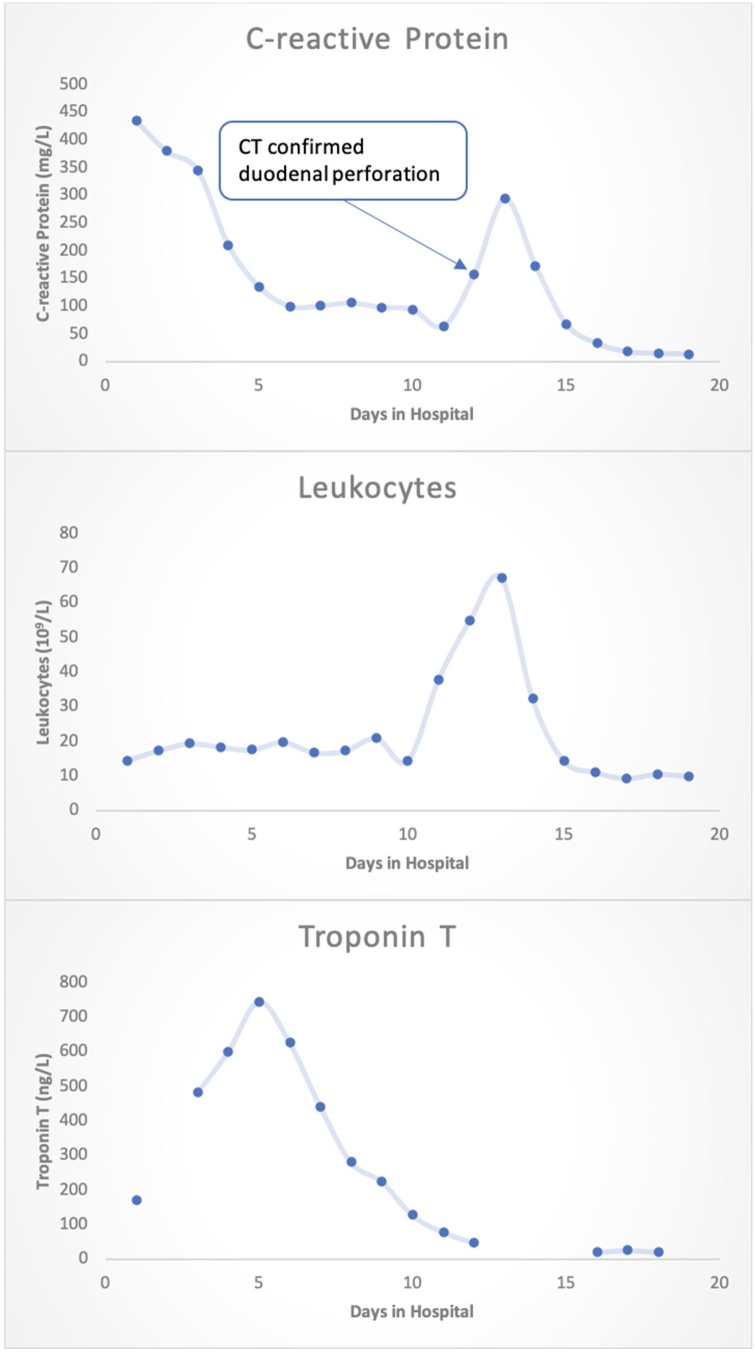
Laboratory development throughout the hospital stay. The c-reactive protein (CRP), leukocytes and troponin T throughout the hospital stay. Laboratory values at admission were as follows: Creatinine: 220 μmol/L (ref<100). CRP: 435 mg/L (ref<5). Leukocytes: 14.5 10^9^/L (ref:3.5–5.5). Troponin T: 172 ng/L (ref<5). Lactate: 5 mmol/L (ref<2.3).

**Fig. 4:**
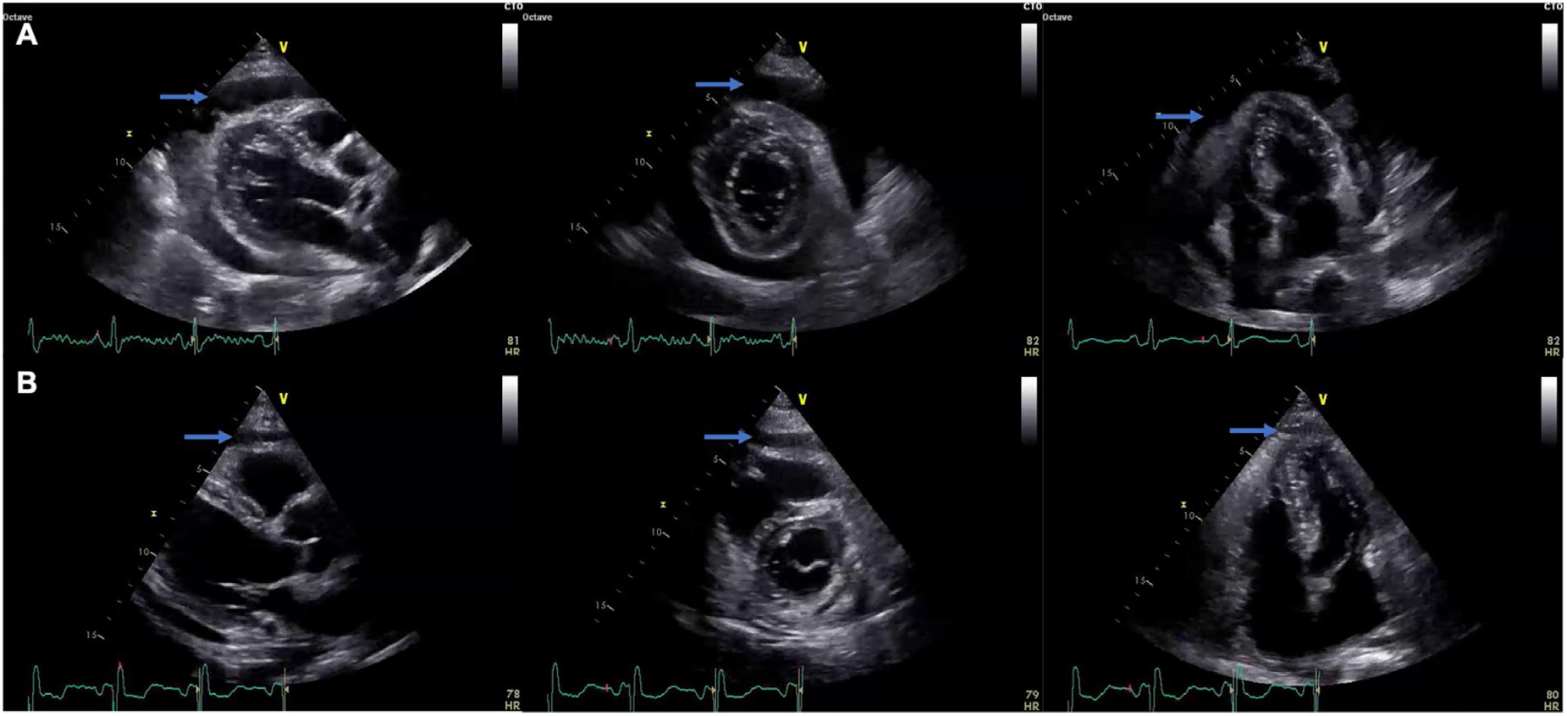
Transthoracic Echocardiograph. A) At the cardiac intensive care unit there were signs of hemodynamic effects with a collapse of the right ventricle in diastole and collapse of the right atrium in systole. The pericardial effusion increased to about 2 cm. There was a high-echogenic structure suspicious for fibrin deposition. B) The pericardiocentesis resolved the hemodynamic instability. The pericardial effusion was culture negative for mycobacteria. Negative PCR for M. tuberculosis-complex-DNA but positive sequencing (16S) for Neisseria meningitidis DNA. Blue arrow pointing towards pericardial effusion.

The patient also exhibited signs of heart failure, such as pulmonary congestion and pitting edema of the legs, and thus received furosemide. Due to an increasing pericardial effusion causing early signs of cardiac tamponade (Fig. 3), the patient received a pericardiocentesis. The pericardial fluid was sent for PCR which was positive for meningococcus DNA. Cortisone and a proton pump inhibitor were administered to reduce complications of inflammation. The following day the patient developed abdominal pain. A CT-scan showed signs of intestinal perforation and the patient was switched to meropenem antibiotics and brought down for emergency surgery. The explorative surgery found a 2.5 cm large perforation by the duodenal bulb. The patient received stitches, an Omentpatch, and drainage. He was empirically placed on helicobacter pylori eradication therapy. Fig. 4 illustrates how c-reactive protein (CRP), leukocytes and Troponin T varied over the course of the hospital stay.

At three-month follow-up, the patient had just finished his colchicine therapy. The transthoracic ultrasound showed a slight dilatation of the ascending aorta, a slight insufficiency of the tricuspid and mitral valves, but was otherwise normal. The patient had to a large extent recovered from his illness.

## Discussion

This patient presented with retrosternal chest pain in the absence of clear clinical signs of infection. The EKG showed generalized ST-elevation and a presumed new right bundle branch block. Therefore, while awaiting the blood results, the patient was rushed to the angiography lab in order to explore a potential cardiac event. The results of the initial investigations ultimately pointed towards myopericarditis and disseminated meningococcal disease.

The presentation of MenW meningococcemia is known to be atypical with symptoms varying from pneumonia, septic arthritis, endocarditis, epiglottitis, and gastrointestinal symptoms [9]. This highlights the difficulty of recognizing the correct diagnosis based solely on clinical findings. Furthermore, due to the possibility of disease transmission, the patient’s next of kin received ciprofloxacin antibiotics prophylactically, as recommended by concurrent guidelines [10].

This patient also, unfortunately, experienced perforation of the duodenum. Critically ill patients have an increased risk for gastrointestinal bleeding, which can in part be prevented by the use of proton pump inhibitors [11,12]. It is plausible that delaying the use of cortisone and earlier intervention with a PPI could have avoided the perforation in our case.

## Conclusions

This report emphasizes the importance of keeping a broad differential diagnosis on an elderly patient presenting with chest pain to the E.R. The patient did not present with classic signs of infection and had it not been for the laboratory values being elevated, the patient’s blood cultures may have been delayed. Moreover, it is worth considering a proton pump inhibitor on a patient experiencing a critical illness.

## Funding

No funding was received for this work.

## Ethical approval

We have not asked for IRB approval for this case report.

## Consent

Verbal and written informed consent were obtained from the patient for publication of this case report and accompanying images. A copy of the written consent is available for review by the Editor-in-Chief of this journal on request.

## Author statement

Michael Platten: Conceptualized study. Curated Data. Investigation. Formal Analysis. Methodology. Wrote manuscript.

Layth Aladellie: Conceptualized study. Curated Data. Investigation. Supervised. Critically reviewed and edited manuscript.

## Declaration of competing interest

The authors report no declarations of interest.
